# Professional Quality of Life Among Physicians and Nurses Working in Portuguese Hospitals During the Third Wave of the COVID-19 Pandemic

**DOI:** 10.3389/fpsyg.2022.814109

**Published:** 2022-01-31

**Authors:** Carla Serrão, Vera Martins, Carla Ribeiro, Paulo Maia, Rita Pinho, Andreia Teixeira, Luísa Castro, Ivone Duarte

**Affiliations:** ^1^School of Education, Polytechnic of Porto, Porto, Portugal; ^2^Center for Research and Innovation in Education (inED), Porto, Portugal; ^3^Center for Health Technology and Services Research, Faculty of Medicine, University of Porto, Porto, Portugal; ^4^Pulmonology Department, Centro Hospitalar de Vila Nova de Gaia/Espinho, Vila Nova de Gaia, Portugal; ^5^Instituto Ciências Biomédicas Abel Salazar, Universidade do Porto e CHUPorto, Porto, Portugal; ^6^Department of Community Medicine, Information and Health Decision Sciences, Faculty of Medicine, University of Porto, Porto, Portugal; ^7^ADiT-LAB, Instituto Politécnico de Viana do Castelo, Viana do Castelo, Portugal; ^8^School of Health, Polytechnic of Porto, Porto, Portugal

**Keywords:** COVID-19, professional quality of life (ProQOL-5), compassion satisfaction, compassion fatigue, burnout, secondary traumatic stress, nurses, physicians

## Abstract

**Background:**

In the last 2 weeks of January 2021, Portugal was the worst country in the world in incidence of infections and deaths due to COVID-19. As a result, the pressure on the healthcare system increased exponentially, exceeding its capacities and leaving hospitals in near collapse. This scenario caused multiple constraints, particularly for hospital medical staff. Previous studies conducted at different moments during the pandemic reported that COVID-19 has had significant negative impacts on healthcare workers’ psychological health, including stress, anxiety, depression, burnout, post-traumatic stress symptoms, and sleep disturbances. However, there are many uncertainties regarding the professional quality of life of hospital nurses and physicians. To address gaps in previous research on secondary traumatic stress, we focused on healthcare workers working in hospitals affected by a major traumatic event: the third wave of COVID-19.

**Objectives:**

The aim of the present study was to identify the contribution of personal and work-related contextual variables (gender, age, parental status, occupation, years of experience, working with patients affected by COVID-19) on professional quality of life of healthcare workers.

**Methods:**

Cross-sectional study with a web-based questionnaire given to physicians and nurses working in a hospital setting. A total of 853 healthcare professionals (276 physicians and 586 nurses; median age 37 years old) participated in the survey assessing professional quality of life compassion satisfaction, secondary traumatic stress, and burnout. Factors of professional quality of life were assessed using regression analysis.

**Results:**

Most of the participants showed moderate (80%; *n* = 684) or high (18%; *n* = 155) levels of compassion satisfaction, whereas the majority of them experienced moderate levels of burnout (72%; *n* = 613) and secondary traumatic stress (69%; *n* = 592). The analyzed variables demonstrated no differences between professionals who were directly or not involved in the care of COVID-19 patients. Parental status was found to be a significant factor in compassion satisfaction. Female gender was significantly associated with more susceptibility to secondary traumatization. Factors that may potentially contribute to burnout include years of professional experience and the number of work hours per week.

**Conclusion:**

The COVID-19 pandemic has created a new challenge for the healthcare system. Burnout and secondary traumatic stress can lead to medical errors and impact standards of patient care, particularly compromising compassionate care. It is therefore recommended that hospitals develop psychoeducational initiatives to support professionals in dealing with barriers to compassion.

## Introduction

By January 2021, the World Health Organization (WHO) was reporting more than 98.2 million reported cases and over 2.1 million deaths globally since the start of the COVID-19 pandemic (World Health Organization [WHO], 2021). Portugal experienced its third wave of surging numbers of COVID-19 patients in early January 2021; consequently, the government imposed new, stricter safety measures on January 19. In Portugal, the peak of the third wave of the pandemic was reached on January 29. By this date there were 179,939 active cases, 6,544 hospitalized patients, and 843 patients in intensive care units (da Saúde, 2021). In the last 2 weeks of January, Portugal recorded the highest COVID-19 infection rate in the European Union: 1,429.43 per 100,000 inhabitants (European Center for Disease Prevention and Control [ECDC], 2021). During this period, Portugal had the highest number per capita of new infections and deaths from COVID-19 in the world. This increased the pressure on intensive care units exponentially, surpassing their capacities and leaving hospitals in near collapse (Demony, 2021). This scenario caused multiple issues, particularly for hospital medical staff, such as increased demand for healthcare, increased patient mortality, overwhelming workload, working with patients infected with COVID-19 (Du et al., 2020; Duarte et al., 2020; Lai et al., 2020), emotional overburden, and uncertainty (Spoorthy, 2020). In addition, medical staff from various specialties had to relocate to assist COVID-19 patients (Vieta et al., 2020). Furthermore, healthcare workers were exposed to a high level of patient suffering, and had to deal with patients’ traumatic experiences (Brooks et al., 2020) and the unexpected loss of friends, family, and colleagues in an overwhelmed healthcare system. All of these experiences have the potential to affect professional quality of life.

Previous studies have reported that COVID-19 has had significant negative impacts on healthcare workers’ psychological health, including stress, anxiety, depression (Duarte et al., 2020; Lai et al., 2020; Weilenmann et al., 2020; Chirico and Magnavita, 2021), burnout (Duarte et al., 2020; Khasne et al., 2020; Weilenmann et al., 2020; Lee et al., 2021; Serrão et al., 2021), post-traumatic stress symptoms (Arnetz et al., 2020; Luceño-Moreno et al., 2020), and sleep disturbance (Chirico and Magnavita, 2021). For example, in a study conducted by Duarte et al. (2020) during the first wave, more than half of the 2,008 participating Portuguese healthcare workers experienced high levels of work-related burnout, and more than 30% were found to suffer from anxiety, stress, and depression. Likewise, a study in Spain, one of the top five countries with the highest number of people infected, confirmed that 58.6% (*n* = 833) of health workers had a possible anxiety disorder, 46% (*n* = 648) suffered from depressive symptoms, and 56.6% (*n* = 805) presented symptoms of post-traumatic stress disorder (Luceño-Moreno et al., 2020). The symptoms of post-traumatic stress disorder were more severe when associated with the variables “hospital staff professionals,” “female sex,” and “likelihood of being infected with COVID-19.” These findings contrast with a study in China (Zhang et al., 2020), where the variables “female sex” and “working on the frontline” were not predictive of high levels of post-traumatic stress disorder.

Another consequence of the COVID-19 outbreak is secondary traumatic stress. In this sense, other variables such as “secondary traumatic stress” and “compassion satisfaction” become important for a clearer understanding of the impact of COVID-19 on health professionals. Nevertheless, these constructs have hardly been investigated during this pandemic.

Compassion satisfaction, secondary traumatic stress, and burnout are dimensions of the construct of professional quality of life (Figley, 1995). The quality of life of the professional providing care to those who have experienced trauma is a recent area of interest among researchers (Stamm, 2010), and their secondary exposure to trauma is an essential pathway to be studied. Professional quality of life is the quality that an individual feels and derives from his or her work (Stamm, 2010). This quality incorporates negative and positive aspects (Figley, 1995). The negative aspects of this construct are explained through the concept of compassion fatigue, which includes two dimensions: secondary traumatic stress and burnout. Secondary traumatic stress refers to the negative feeling resulting from fear or work-related trauma due to secondary exposure to people who have experienced traumatic or extremely stressful events, manifested by feelings of fear, sleep difficulties, intrusive images related to patients’ traumatic experiences, or avoidance of anything that may recall such experiences (Figley, 1995). Burnout manifests through feelings of anger, frustration, sadness, discontentment, exhaustion, and depression and is related to feeling that one’s efforts do not make a difference, overwork and difficulties coping with pressure, or little support in the work environment (Stamm, 2010). Burnout is defined as a response to prolonged exposure to demanding interpersonal situations and is characterized by “emotional exhaustion, depersonalization, and reduced personal accomplishment” (Maslach et al., 2002). Compassion fatigue is defined as the formal caregiver’s reduced capacity or interest in being empathic or “bearing the suffering of clients” and consists of “the natural consequent behaviors and emotions resulting from knowing about a traumatizing event experienced or suffered by a person” (Figley, 1995, p. 7). It is the result of prolonged exposure to secondary traumatic stress derived from contact with patients and is facilitated by issues derived from the work environment.

The positive aspects of professional quality of life are explained through the concept of compassion satisfaction, which is defined as the pleasure derived from the opportunity to do a good job associated with feelings of accomplishment, fulfillment, or work-related satisfaction (Stamm, 2005). It can be a feeling of pleasure resulting from being able to help others or positive feelings that come from being part of something constructive in the workplace or a greater good at the societal level. Stamm (2010) suggests that this dimension acts as a factor that protects the professional from stressors at work.

In 2018, a meta-analysis including 21 studies on nurses detected high rates of compassion satisfaction (47.6%), compassion fatigue (52.6%), and burnout (52%) (Zhang et al., 2018a). In this regard, the authors warn that these professionals are especially prone to reduced compassion satisfaction, higher compassion fatigue, and burnout when they experience repeated exposure to situations with a high emotional and physical load originating from caring for patients, most of whom are seriously ill and many of whom are traumatized.

In a 2020 study involving 506 healthcare professionals from Spain, 94% showed medium to high compassion fatigue (depending on the version—secondary traumatic stress), 84% showed medium to high burnout, and 84.4% of the total sample experienced compassion satisfaction (Ruiz-Fernández et al., 2020). Secondary traumatic stress and burnout were more severe in physicians than in nurses. Furthermore, nurses scored higher on compassion satisfaction. In another Spanish survey of 973 healthcare professionals, 90.6% showed high levels of compassion satisfaction, highlighting a higher prevalence of this variable among people in the 35–55-year age range, as well as among more technicians than nurses, although nurses surpassed physicians (Dosil et al., 2020).

Unfortunately, burnout and secondary traumatic stress can lead to medical errors and impact standards of patient care, particularly compromising compassionate care (e.g., Dewar, 2013). In addition, levels of burnout and secondary traumatic stress can also affect relationships with coworkers and lead to physical and mental health conditions (Cocker and Joss, 2016).

Regardless of the effects of COVID-19, based on these findings, it is clear that healthcare workers must be prepared to deal with possible psychological and work-related consequences. Thus, this issue deserves particular attention in the context of a pandemic. These studies took place at different moments on the pandemic curve; however, none assessed compassion fatigue, burnout, and professional satisfaction in the third wave of a prolonged struggle against COVID-19.

To address gaps in previous research on compassion fatigue, we focused on healthcare workers working in hospitals affected by a major traumatic event: the third wave of COVID-19. The primary aim of the present study was to identify the contribution of personal and professional contextual variables (gender, age, marital status, working position, years of experience, working or not with patients affected by COVID-19) on professional quality of life—satisfaction compassion, burnout, and secondary traumatic stress—of physicians and nurses, during the third wave of COVID-19 in Portugal, using linear regression. As a secondary aim, a comparison between healthcare workers directly involved in caring for COVID-19 patients and those who were not was performed with regard to personal and professional contextual variables, compassion satisfaction, burnout, and secondary traumatic stress.

## Materials and Methods

### Participants

A total of 853 healthcare professionals (586 nurses and 267 physicians) working in hospital units participated in the study, with a median age of 37 (31; 46) years old. The participants were divided into seven regions based on the Portuguese Territorial Units for Statistics Level II (NUTS II): North (371 participants; 43.5%), Center (210 participants; 24.6%), Lisbon (124 participants; 14.5%), Alentejo (18 participants; 2.1%), Algarve (21 participants; 2.5%), Autonomous Region of the Azores (83 participants; 9.7%), and Autonomous Region of Madeira (26 participants; 3%).

### Measures and Instruments

Sociodemographic and vocational contextual variables were collected using a self-administered online questionnaire. Professional quality of life was evaluated with the Professional Quality of Life Scale—Version 5 (ProQOL-5; Stamm, 2009; Portuguese version by Duarte, 2017), consisting of 30 items with a Likert-type scale of 5 points (1 = Never 2 = Rarely 3 = Sometimes 4 = Often 5 = Very Often). This scale is composed of three subscales: compassion satisfaction, burnout, and secondary traumatic stress. Respondents were instructed to indicate how frequently they had experienced each item in the previous 30 days. The questionnaires were scored by summing the item responses for each 10-item subscale, with higher scores indicating higher levels of compassion satisfaction, secondary traumatic stress, and burnout. The scores from each of the subscales can be categorized into secondary traumatic stress, compassion satisfaction, and burnout: < 22 low; 23–41 medium; > 42 high. The Portuguese version of the ProQOL-5 has a Cronbach’s alpha of 0.88 for compassion satisfaction, 0.82 for secondary traumatic stress, and 0.86 for burnout (Duarte, 2017). The Cronbach’s alpha of the current study was 0.88 for compassion satisfaction, 0.81 for secondary traumatic stress, and 0.79 for burnout.

### Procedure

Data collection took place from March 1 to May 2, 2021 (the last day of the state of emergency). A questionnaire created using the Google^®^ Forms platform was made available to participants via a link shared through direct e-mail and social networks (Facebook, Instagram, Linked In, and WhatsApp) following a snowball approach. Additionally, different professional networks and companies were contacted to share the questionnaires. There was no missing data because all questions were mandatory, meaning that only completed questionnaires were collected.

Ethical procedures in accordance with the Declaration of Helsinki were accomplished via analysis and approval of the study by the Ethics Committee of São João Hospital Center (Ref 65/2021 on February 19, 2021). All respondents provided informed consent prior to accessing the questionnaires.

### Data Analysis

Data was exported from Google^®^ Forms to a Microsoft Excel^®^ 2016 (United States) spreadsheet and all statistical analyses were performed using SPSS Statistics (version 26.0; SPSS^®^ Inc., Chicago, Illinois, United States) and Jamovi 1.1.9.0 (datalab.CC, Sidney, Australia).

Categorical variables were described by absolute and relative frequencies, n (%). The normality of quantitative variables was verified by visual analysis of histograms, and those variables with assumed normal distributions were summarized by their mean and standard deviation (SD). Otherwise, quantitative variables were described by their median (Med) and interquartile interval [Q_1_; Q_3_].

Differences between participants were analyzed using Mann-Whitney *U*-tests for quantitative non-normally distributed data, and chi-squared tests were used for categorical data. Pearson’s correlation coefficient (r) was used to access the correlation between normally distributed quantitative variables. Cronbach’s alpha was computed to assess the internal consistency of each subscale, and a value above 0.7 was considered acceptable (Kline, 2010).

For each outcome—compassion satisfaction, burnout, and secondary traumatic stress—a univariate multiple linear regression analysis was performed. In each multiple regression, the independent variables to be included were chosen by performing simple linear regressions with each variable in the dataset, including sociodemographic, professional (the working hours variable was codified into the following categories: Group 1 - ≤ 40 h; Group 2 - > 40 and ≤ 55 h, Group 3 - > 55 working hours for physicians and Group 1 - ≤ 35 h; Group 2 - > 35 and ≤ 50 h, Group 3 - > 50 working hours for nurses), and COVID-19-related variables. These results can be found in more detail in the Online Supporting Material. All variables correlating with the outcomes at *p* ≤ 0.2 in the simple regression were included in the final linear regression models. Then, non-significant independent variables were removed, one at a time, in descending order of their *p*-values. Only the significant variables were maintained in the multiple models for compassion satisfaction, burnout, and secondary traumatic stress. The results of the linear regressions are presented with unstandardized coefficient values (β), 95% confidence intervals (95% CIs), and *p*-values. The final models were evaluated using the coefficients of determination (*R*^2^), the F statistic of the overall model test, and respective *p*-values. The assumptions of the linear regression models were verified as follows: (a) visual analysis of histograms to verify the normality of residuals; (b) *t*-test to determine whether the mean of the residuals was equal to zero; and (c) visual analysis of residuals plots vs. the fitted predictive values to check for homoscedasticity. Values of *p* ≤ 0.05 were considered significant.

## Results

### Sample Characteristics of Participants

A total of 853 physicians and nurses working in Portuguese hospitals during the COVID-19 pandemic completed the questionnaire. Given the sample size of 853 participants, for a confidence level of 95%, and assuming the most conservative scenario of 50% estimated proportion, a margin of error of 3.3% is estimated for the proportions computed. At the time of the study, most of the sample worked in public hospitals (*n* = 832; 97.5%), were permanent employees (*n* = 780; 91.4%), and worked day shifts with nights or on-call (*n* = 568; 66.6%). Four hundred and forty-six healthcare workers (52.3%) were directly involved in caring for COVID-19 patients (i.e., intensive care units, infectious disease, pulmonary medicine, and internal medicine wards), while 407 (47.7%) were involved in different units (i.e., oncology, pediatric, and psychiatric wards). Approximately 85% indicated that they had already taken the COVID-19 vaccine. The characteristics of the participants are summarized in Table 1.

**TABLE 1:** Demographic and professional characteristics (*n* = 853).

Characteristics/Variables	Total (*n* = 853)	Directly involved in caring for COVID-19 patients (*n* = 446, 52.3%)	Not directly involved in caring for COVID-19 patients (*n* = 407, 47.7%)	*P*-value
Age (years), Med [Q_1_; Q_3_]	37 [31; 46]	37 [30; 44]	38 [32; 47]	0.007^a^*
Gender, n (%)				0.320^b^
Female	693 (81.2)	368 (53.1)	325 (46.9)	
Male	160 (18.8)	78 (48.8)	82 (51.2)	
Occupation, n (%)				0.004^b^*
Nurse	586 (68.7)	326 (55.6)	260 (44.4)	
Physician	267 (31.3)	120 (44.9)	147 (55.1)	
Marital status, n (%)				0.508^b^
Married or non-marital partnership	493 (57.8)	253 (51.3)	240 (48.7)	
Single or divorced or widowed	360 (42.2)	193 (53.6)	167 (46.4)	
Parents, n (%)				0.260^b^
No	428 (50.2)	232 (54.2)	196 (45.8)	
Yes	425 (49.8)	214 (50.4)	211 (49.6)	
Education level, n (%)				0.110^c^
Graduate and post-graduate	536 (62.8)	290 (54.1)	246 (45.9)	
Master	286 (33.5)	136 (47.6)	150 (52.4)	
Doctorate degree	6 (0.7)	5 (83.3)	1 (16.7)	
Others	25 (2.9)	15 (60.0)	10 (40.0)	
Length of work experience, n (%)				0.011^b^*
Five years or less	185 (21.7)	97 (52.4)	88 (47.6)	
From 6 to 10 years	150 (17.6)	96 (64.0)	54 (36.0)	
From 11 to 15 years	155 (18.2)	79 (51.0)	76 (49.0)	
More than 15 years	363 (42.6)	174 (47.9)	189 (52.1)	
Length of service in current unit, n (%)				0.229^b^
6 months or less	109 (12.8)	61 (56.0)	48 (44.0)	
Between 6 months and 1 year	117 (13.7)	71 (60.7)	46 (39.3)	
Between 2 and 5 years	216 (25.3)	113 (52.3)	103 (47.7)	
Between 6 and 10 years	126 (14.8)	62 (49.2)	64 (50.8)	
Between 11 and 15 years	100 (11.7)	53 (53.0)	47 (47.0)	
More than 15 years	185 (21.7)	86 (46.5)	99 (53.5)	
Working hours per week (*N* = 843), n (%)				
Group 1	296 (35.1)	115 (26.2)	181 (44.8)	<0.001^a^*
Group 2	412 (48.9)	240 (54.7)	172 (42.6)	
Group 3	135 (16.0)	84 (19.1)	51 (12.6)	
Diagnosed health problem, n (%)				0.098^b^
No	637 (74.7)	344 (54.0)	293 (46.0)	
Yes	216 (25.3)	102 (47.2)	114 (52.8)	
Exercises managerial duties, n (%)				0.220^b^
No	764 (89.6)	405 (53.0)	359 (47.0)	
Yes	89 (10.4)	41 (46.1)	48 (53.9)	

^a^Mann Whitney test; ^b^Chi-square test; ^c^Fisher’s exact test. *p < 0.05.

### Results of Professional Quality of Life-5—Compassion Satisfaction, Secondary Traumatic Stress, and Burnout

The average levels of compassion satisfaction, secondary traumatic stress, and burnout among nurses and physicians were separated into low, moderate, and high groups. Moderate levels of compassion satisfaction, burnout, and secondary traumatic stress were found in 684 (80.2%), 613 (71.9%), and 592 (69.4%) participants, respectively. No statistically significant differences were found between healthcare workers who were directly involved in caring for COVID-19 patients and those who were not (Not-COVID) with regard to compassion satisfaction, burnout, and secondary traumatic stress (Table 2). Moreover, results show that burnout exhibits a high negative correlation with compassion satisfaction (*r* = −0.687, *p* < 0.001) and a high positive correlation with secondary traumatic stress (*r* = 0.624, *p* < 0.001), while secondary traumatic stress and compassion satisfaction show a weak negative correlation (*r* = −0.236, *p* < 0.001).

**TABLE 2:** Professional quality of life—COVID or NOT_COVID.

Variables	Variables	COVID		NOT-COVID		*p*-value (Mann-Whitney U)
		Med [Q_1_; Q_3_]	N (%)	Med [Q_1_; Q_3_]	N (%)	
Compassion satisfaction	Low	37 [33; 41]	10 (2.2)	37 [33; 41]	4 (1.0)	0.631
	Medium		356 (79.8)		328 (80.6)	
	High		80 (17.9)		75 (18.4)	
Secondary traumatic stress	Low	26 [22; 29]	133 (29.8)	25 [22; 29]	123 (30.2)	0.424
	Medium		311 (69.7)		281 (69.0)	
	High		2 (0.4)		3 (0.7)	
Burnout	Low	26 [22; 30]	121 (27.1)	25 [22; 29]	116 (28.5)	0.387
	Medium		323 (72.4)		290 (71.3)	
	High		2 (0.4)		1 (0.2)	

### Results of Professional Quality of Life-5—Compassion Satisfaction, Secondary Traumatic Stress, and Burnout: Multivariate Analysis

In the final linear regression model for compassion satisfaction as the outcome variable, the only significant associations were with the variable “parents,” explaining approximately 1.3% of the variability existing in the dependent variable. Health professionals with children have an average of 1.31 more points on the compassion satisfaction scale. For secondary traumatic stress as the outcome variable, only the variable “gender” was significantly associated with the outcome, explaining approximately 2.4% of the variability existing in the dependent variable. Female health professionals scored an average of 2.33 more points on the scale of secondary traumatic stress than males. Concerning the final multiple linear regression model, with burnout scale as the outcome variable, the variables “work experience” and “working hours” were significantly associated with the outcome, explaining approximately 1.9% of the variability existing in the dependent variable. Health professionals with “6–10 years” of experience show on average 1.33 points more on the scale of burnout than those with “5 years or less” of professional experience. Moreover, professionals within Group 3 (physicians working > 50 h and nurses working > 55 h per week) scored, on average, 1.72 points higher on the burnout scale than professionals in Group 1 (physicians working ≤ 40 h and nurses working ≤ 35 h per week) (Table 3).

**TABLE 3:** Regression coefficients for professional quality of life subscales as outcomes and socio-demographic, and professional variables as predictors from univariate simple and multiple linear regressions.

Variables	Compassion satisfaction β [95% CI]	Secondary traumatic stress β [95% CI]	Burnout β [95% CI]
Parents			
No	*Reference*		
Yes	1.31 [0.53; 2.09] ***		
Gender			
Male		*Reference*	
Female		2.33 [1.33; 3.33]***	
Work experience			
Five years or less			*Reference*
From 6 to 10 years			1.33 [0.06; 2.59]*
From 11 to 15 years			−0.06 [−1.31; 1.19]
More than 15 years			−0.07 [−1.11; 0.97]
Working hours			
Group 1			*Reference*
Group 2			0.67 [−0.20; 1.54]
Group 3			1.72 [0.53; 2.91]**
*R* ^2^	0.0126	0.024	0.0194
*F*	10.8***	20.9***	3.30**

*CI, confidence interval.*

*p ≤ 0.05; **p ≤ 0.01; ***p ≤ 0.001.

## Discussion

This research aimed to assess professional quality of life among Portuguese healthcare workers (nurses and physicians) working in hospitals during the first months of 2021. In January and February, Portugal endured a third wave of the COVID-19 pandemic, and the national healthcare system faced possible collapse. As such, healthcare professionals are likely to experience compassion fatigue (burnout and secondary traumatic stress) triggered by the recurrent practice of empathy, prolonged exposure to secondary trauma, and the work environment (Cavanagh et al., 2020). In addition, limited resources and multiple healthcare demands during this period may also contribute to the risk of developing compassion fatigue.

Our findings show that healthcare workers exposed to the third wave of the COVID-19 pandemic presented both positive and negative psychological outcomes. The results revealed a moderate prevalence of burnout (72%) and secondary traumatic stress (69%), and moderate (80%) to high (18%) levels of compassion satisfaction. Similar findings were seen in Ecuador during the first wave of the COVID-19 pandemic, where healthcare professionals reported medium levels of burnout and compassion fatigue (Cuartero-Castañer et al., 2021). However, Cuartero-Castañer et al. (2021) found high levels of compassion satisfaction and argue that these results may have been due to strengthened recognition of the healthcare task force. Another study conducted during the first wave in Spain by Ruiz-Fernández et al. (2020) found that the majority of healthcare professionals had high levels of compassion fatigue and medium to high levels of burnout and compassion satisfaction. In contrast, Trumello et al. (2020) found that professionals working in areas with higher levels of contagion presented higher levels of burnout and lower levels of compassion satisfaction, which contradict our results. The results we achieved may be related with the fact that, by the third wave, the professionals could have developed mechanisms (such as resilience) to protect themselves, which may act as a protective factor during their contact with the COVID-19 pandemic (Serrão et al., 2021). Another possible explanation for this result may be that most of our sample (85%) had already been vaccinated against COVID-19. According to Abdelghani et al. (2020) study, the perceived fear of COVID-19 virus infection was positively correlated with burnout emotional exhaustion, and depersonalization symptoms.

Obviously, these results must be interpreted with caution, and the timing and the countries where the studies were conducted must be kept in consideration. During wave peaks and in countries that reported higher COVID-19 infection rates per 100,000 inhabitants, hospitals were overcrowded and healthcare professionals were exposed to much more pressure and stress. Another important aspect is the pre-COVID-19 state of exhaustion of healthcare workers. A Portuguese study from 2016 (Marôco et al., 2016) concluded that 44 percent of physicians and fifty percent of nurses demonstrated high levels of burnout, which could influence the levels of burnout and fatigue compassion observed in healthcare workers during COVID-19 pandemic.

Moreover, results show that burnout exhibits a high negative correlation with compassion satisfaction and a high positive correlation with secondary traumatic stress, while secondary traumatic stress and compassion satisfaction show a weak negative correlation. These associations have been mentioned by other authors (Ortega-Galán et al., 2020; Ruiz-Fernández et al., 2020; Cuartero-Castañer et al., 2021; Lee et al., 2021) and could indicate that compassion satisfaction may be acting as a protective variable against burnout and secondary traumatic stress. In fact, the balance between those variables determines the degree of professional quality of life, so as observed in previous research, this relationship between the results was expected (Wee and Myers, 2003; Buselli et al., 2020; Dosil et al., 2020).

Compassion satisfaction is the capacity to receive consideration from providing care and is related with self-efficacy at work and more adequate coping mechanisms (Cuartero-Castañer et al., 2021). In our study, compassion satisfaction was higher in healthcare professionals who were parents compared to those who were not. This could be explained by the fact that professionals who have children may have developed coping mechanisms and often transfer their care toward patients since they are frequently away from their children and family.

The results showed that nurses experienced higher levels of compassion satisfaction than doctors, although these differences were not statistically significant. Our expectations were to obtain results similar to previous research (e.g., Buselli et al., 2020; Ruiz-Fernández et al., 2020). For example, Ruiz-Fernández et al. (2020) found that nurses were shown to have significant scores of compassion satisfaction compared to physicians. These results contradict those obtained by Buselli et al. (2020) who found that, compared to being a nurse, being a doctor had a positive impact on compassion satisfaction, probably related with the perception of personal success regarding the effects of the prescribed treatments and the feeling of “mission accomplished.”

Health professionals who had been in direct contact with COVID-19 patients had higher levels of compassion satisfaction, secondary traumatic stress, and burnout than those who had not, although the differences were not statistically significant. These results are in line with previous studies (Dosil et al., 2020; Zhang et al., 2020) and suggest that, in hospital settings, both groups could face identical threats to COVID-19. However, this contradicts the results of previous studies showing poor mental health outcomes in professionals who directly care for and treat COVID-19 patients (Ruiz-Fernández et al., 2020; Trumello et al., 2020). Another interesting result was obtained by Li et al. (2020) and Wu et al. (2020), who found that healthcare workers who worked with COVID-19 patients had lower levels of secondary traumatic stress and burnout, respectively. The authors defended that these professionals have developed mechanisms of self and emotional control and are probably better informed about the evolution of the infection compared with those working in non-COVID-19 wards. This result may also be due to the particular characteristics of the health systems of different countries.

Our findings show that female healthcare professionals present a higher level of secondary traumatic stress than males, which is in line with previous research (Buselli et al., 2020; Luceño-Moreno et al., 2020; Ortega-Galán et al., 2020; Cuartero-Castañer et al., 2021). This could be related with the roles that women play in society both as professionals and at home (Duarte et al., 2020; Ortega-Galán et al., 2020), and these roles may have been more difficult to manage during the pandemic given the demands imposed on both. There was a lockdown during the third wave of COVID-19 in Portugal, which made it difficult for female healthcare professionals to balance caring for their homes and children with the demands of their workplace (Duarte et al., 2020). On the other hand, Hernández-Padilla et al. (2020) draw attention to the fact that being a female healthcare professional is associated with higher levels of stress and compassion fatigue because the female gender is the most prevalent gender among healthcare workers.

Our results show that healthcare professionals with 5–10 years of professional experience presented higher risk of burnout compared with professionals with less years of experience. This result can be justified by the fact that this period is for both nurses and physicians, the first years of practice as a specialist. These results are in line with previous research (e.g., Baptista et al., 2021).

Additionally, as working hours increased, so did burnout levels, which was expected. This finding was similar to the results presented by Weilenmann et al. (2020), where 40% of professionals said they were working more hours than before the COVID-19 pandemic. This should be a concern for employers, as burnout could lead to more fatigue, exhaustion, and reduced attention, which could lead to a higher number of adverse events and a reduced perceived quality of patient care (Kakemam et al., 2021) and absenteeism.

There is no doubt that the COVID-19 pandemic has had a profound impact on health services and health workers (e.g., Serrão et al., 2021; Weilenmann et al., 2021). Although many of the effects will only become apparent in the medium or long term, the available data highlights the need for psychoeducational initiatives and strategies to foster a compassionate organizational culture.

Although the concept of compassion fatigue is the construct that underlies the ProQOL instrument and has been extensively researched, several authors have raised criticisms in recent years regarding this denomination (e.g., Klimecki et al., 2014; Sinclair et al., 2016; Vrtička et al., 2017; Hofmeyer et al., 2019), proposing the alternative concepts of “empathy fatigue” or “empathic distress fatigue.” Although empathy and compassion are important social skills for the caregiving process, empathy is a socio-affective response and “the ability to ‘feel with’ others when we are exposed to their distress and suffering” (Hofmeyer et al., 2020). In turn, compassion is a socio-cognitive response and a “‘feeling for’ others who are in pain with warmth” (Hofmeyer et al., 2020). Neuroscience studies using functional magnetic resonance imaging (fMRI) support this proposal, concluding that empathy and compassion are associated with two different networks at the brain level (e.g., Klimecki et al., 2014; Vrtička et al., 2017). These important findings have shown that empathizing with the pain of others activates the same parts of the brain that are involved in self-pain processing (Klimecki et al., 2014). In this sense, and according to the authors, although “empathy is crucial for successful social interactions, excessive sharing of others’ negative emotions may be maladaptive and constitute a source of burnout.” Alternatively, an adaptive response to the suffering of others may involve developing compassion. Having compassion “increased activations in brain networks related to reward, affiliation” (Klimecki et al., 2014) and serves as a buffer against empathic distress fatigue.

Investing in programs that reduce empathic distress fatigue may decrease the high turnover rates seen among physicians and nurses, thereby improving quality of care (Duarte et al., 2016; Zhang et al., 2018b).

These results allow us to outline some recommendations to enhance compassionate care in health systems (Dewar et al., 2011; Dewar, 2013; Sinclair et al., 2016; Neff and Germer, 2018; Tehranineshat et al., 2019). As Tehranineshat et al. (2019) have described so well, compassionate care is a complex and multidimensional construct that integrates ethical, humanistic, spiritual, and communication dimensions. Compassion is an “ethical principle of care among healthcare professionals in order to provide high quality care” (Tehranineshat et al., 2019). However, the ability of healthcare professionals to perform in accordance with this ethical principle may be hindered by the levels of burnout and secondary traumatic stress experienced (Dev et al., 2018). In their systematic review, Dev et al. (2018) state that greater compassion fatigue (burnout and secondary traumatic stress) “predicted greater barriers to compassion” and this scenario may “have consequences for (and be reflected in) how” health workers experience their work context, their involvement with patients, and their professional responsibilities.

Training compassion, according to functional neural plasticity results, “may reflect a new coping strategy to overcome empathic distress and strengthen resilience” (Klimecki et al., 2014).

One possibility for fostering a compassionate organizational culture is to provide professionals with mindfulness self-compassion (MSC) programs (Neff, 2012; Duarte et al., 2016). Self-compassion, a core feature of Buddhist traditions, has recently been introduced into Western psychology. According to Neff (2012), it comprises three interconnected components: “Self-kindness (treating oneself with warmth, respect, and care), common humanity (understanding that being human inherently includes the experience of pain, imperfection, and difficulty), and Mindfulness (clear and balanced awareness of one’s present moment experience of suffering).” MSC programs help develop compassion for self and others (Neff and Germer, 2018). Self-compassion “enhances the perspective-taking skills” (Neff, 2012), thus optimizing the ability to be compassionate with others (e.g., Duarte et al., 2016).

Several studies conducted in recent years on mindfulness-based programs offered to healthcare professionals have shown that these appear to affect a range of mental health outcomes, including anxiety, depression (e.g., Johnson et al., 2015), burnout (e.g., Martín-Asuero et al., 2014), compassion, self-compassion (e.g., West et al., 2014; Raab et al., 2015), and well-being (e.g., West et al., 2014).

These promising results highlight the advantage of investing in the capacities and resources of healthcare teams with a view to foster a compassionate organizational culture.

### Limitations

While contributing empirically to the discussion regarding professional quality of life among Portuguese physicians and nurses working in hospitals, this study has a few limitations. First, the research was conducted online, which might be affected by self-selection bias (Wright, 2005). Second, a convenience sample was collected, which may not adequately represent the population and therefore does not permit extrapolation of the results to other contexts. Third, this study was cross-sectional in design, wherein the phenomenon of professional quality of life was assessed at a single point in time and carried out in the specific timeframe and context of the COVID-19 pandemic, so the data must be interpreted with caution. Four, the sample was recruited from different regions, that could have different infective incidence of the pandemic. Moreover, the sample was not homogeneous for gender. Finally, mental health variables (e.g., depression, anxiety, post-traumatic stress symptoms related to the pandemic) that could affect professional quality of life, as well as previous mental health disorders, were not evaluated.

Furthermore, this study suggests that there may be other variables that can shed light on professional quality of life. In this sense, it would be useful to consider, for example, personal coping strategies and work, client, and personal environments in future research (Stamm, 2010).

Finally, it would be interesting to extend this study to primary healthcare workers, given that Ortega-Galán et al. (2020) found that fatigue compassion was higher in primary care workers than in hospital care workers. Primary care centers are the core of the Portuguese National Health System and primary healthcare workers, too, must face excessive work and patient contact on a daily basis.

## Conclusion

About 70% of Portuguese physicians and nurses were experiencing moderate likelihood of burnout and secondary traumatic stress based on ProQOL scoring. Additionally, their levels of compassion satisfaction were moderate or high. Factors that potentially contribute to the level of burnout of healthcare professional’s include professional contextual variables (years of experience and working hours). Gender and having children were found to be a potential predictor of secondary traumatic stress, and compassion satisfaction, respectively. Besides, no statistically significant differences were found between healthcare workers who were directly involved in caring for COVID-19 patients and those who were not, with regard to professional quality of life levels. These data can be positive, since a professional who has both compassion fatigue and compassion satisfaction may have a better chance of overcoming compassion fatigue and improving job satisfaction. Specific programs to alleviate mental health issues should be provided, particularly to assess the effect of mindfulness-based programs on increasing psychological resources and more adaptive responses to the work environment. More studies are needed in the medium and long term to determine the real impact of the COVID-19 pandemic.

## Data Availability Statement

The raw data supporting the conclusions of this article will be made available by the authors, without undue reservation.

## Ethics Statement

The studies involving human participants were reviewed and approved by the Ethics Committee of São João Hospital Center (Ref 65/2021 on February 19, 2021). The patients/participants provided their written informed consent to participate in this study.

## Author Contributions

CS and ID contributed to conception and design of the study, project administration—supervision, and coordination. LC and AT organized the database and performed the statistical analysis. CS wrote the first draft of the manuscript, manuscript preparation, manuscript revision, reviewing, editing, and manuscript final version approval. CS, VM, RP, and LC wrote sections of the manuscript. PM provided input for the manuscript. All authors were involved in the data collection, and contributed to manuscript revision, read, and approved the submitted version.

## Conflict of Interest

The authors declare that the research was conducted in the absence of any commercial or financial relationships that could be construed as a potential conflict of interest.

## Publisher’s Note

All claims expressed in this article are solely those of the authors and do not necessarily represent those of their affiliated organizations, or those of the publisher, the editors and the reviewers. Any product that may be evaluated in this article, or claim that may be made by its manufacturer, is not guaranteed or endorsed by the publisher.
