# Supplementary material for: Professional Quality of Life Among Physicians and Nurses Working in Portuguese Hospitals During the Third Wave of the COVID-19 Pandemic
Source: Front Psychol. 2022 Jan 31;13:814109. doi: 10.3389/fpsyg.2022.814109 (PMC8845595; doi:10.3389/fpsyg.2022.814109)
Supplement: Supplementary file 1 [file Table_1.pdf]

Supplementary table *Regression coefficients for professional quality of life subscales as outcomes and socio-demographic, and professional variables as predictors from univariate simple linear regressions*

	**Compassion satisfaction**		**Secondary traumatic stress**		**Burnout**	
	**B [95%CI]**	**p-value**	**B [95%CI]**	**p-value**	**B [95%CI]**	**p-value**
Gender						
Male	*Reference*		*Reference*		*Reference*	
Female	-0.23 [-1.23; 0.78]	0.656	2.33 [1.33; 3.33]	<0.001	0.40 [-0.60; 1.41]	0.429
Occupation						
Nurse	*Reference*		*Reference*		*Reference*	
Physician	-0.61 [-1.45; 0.24]	0.158	-0.55 [-1.40; 0.31]	0.210	0.12 [-0.72; 0.97]	0.775
Parents						
No	*Reference*		*Reference*		*Reference*	
Yes	1.31 [0.53; 2.09]	0.001	0.29 [-0.51; 1.08]	0.479	-0.82 [-1.60; -0.04]	0.041
Work experience						
Five years or less	*Reference*		*Reference*		*Reference*	
From 6 to 10 years	-1.08 [-2.33; 0.17]	0.091	-0.08 [-1.35; 1.19]	0.906	1.46 [0.21; 2.71]	0.022
From 11 to 15 years	0.71 [-0.53; 1.95]	0.261	-0.48 [-1.74; 0.78]	0.455	-0.04 [-1.28; 1.20]	0.946
More than 15 years	1.02 [-0.01; 2.04]	0.052	-0.14 [-1.19; 0.90]	0.789	-0.21 [-1.24; 0.82]	0.692
Length of service in current unit						
6 months or less	*Reference*		*Reference*		*Reference*	
Between 6 months and 1 year	-0.31 [-1.83; 1.21]	0.685	-0.61 [-2.15; 0.93]	0.439	0.75 [-0.77; 2.27]	0.333
Between 2 and 5 years	0.00 [-1.34; 1.34]	0.996	-0.31 [-1.67; 1.04]	0.649	0.66 [-0.68; 2.01]	0.332
Between 6 and 10 years	-0.07 [-1.56; 1.42]	0.926	-0.71 [-2.22; 0.81]	0.360	0.67 [-0.82; 2.17]	0.377
Between 11 and 15 years	1.38 [-0.20; 2.96]	0.087	-1.21 [-2.81; 0.39]	0.138	-0.64 [-2.22; 0.94]	0.427
More than 15 years	1.13 [-0.25; 2.51]	0.109	0.02 [-1.37; 1.42]	0.976	0.07 [-1.31; 1.45]	0.915
COVID						
Directly involved in caring for COVID-19 patients	*Reference*		*Reference*		*Reference*	
Not directly involved in caring for COVID-19 patients	-0.13 [-0.91; 0.66]	0.753	-0.35 [-1.14; 0.44]	0.387	-0.42 [-1.20; 0.36]	0.292
Working Hours						
Group 1	*Reference*		*Reference*		*Reference*	
Group 2	-0.44 [-1.31; 0.42]	0.313	0.29 [-0.59; 1.16]	0.523	0.71 [-0.15; 1.58]	0.107
Group 3	-0.17 [-1.34; 1.01]	0.782	0.53 [-0.67; 1.72]	0.388	1.86 [0.68; 3.04]	0.002
